# Nurse-led home-based detection of cardiac dysfunction by ultrasound: results of the CUMIN pilot study

**DOI:** 10.1093/ehjdh/ztad079

**Published:** 2023-12-12

**Authors:** Jasper Tromp, Chenik Sarra, Bouchahda Nidhal, Ben Messaoud Mejdi, Fourat Zouari, Yoran Hummel, Khadija Mzoughi, Sondes Kraiem, Wafa Fehri, Habib Gamra, Carolyn S P Lam, Alexandre Mebazaa, Faouzi Addad

**Affiliations:** Saw Swee Hock School of Public Health, National University of Singapore & The National University Health System, 12 Science Drive 2, #10-01, Singapore 117549, Singapore; Duke-NUS Medical School, 8 College Rd, Singapore 169857, Singapore; Military Hospital Tunis, Q5PH+896, Tunis, Tunisia; Fattouma Bourguiba University Hospital—Research Laboratory LR12SP16 and University of Monastir, QRCM+4GJ, Monastir, Tunisia; Fattouma Bourguiba University Hospital—Research Laboratory LR12SP16 and University of Monastir, QRCM+4GJ, Monastir, Tunisia; Hannibal Clinic, Rue de la feuille d'Erable - les berges du lac 2, Tunis, Tunisia; Us2.ai, 2 College Rd, #02-00, Singapore 169850, Singapore; Faculty of Medicine of Tunis, Habib Thameur Hospital Tunis & University of Tunis El Manar, Q5PG+CJ7, Rue Ali Ben Ayed, Tunis, Tunisia; Faculty of Medicine of Tunis, Habib Thameur Hospital Tunis & University of Tunis El Manar, Q5PG+CJ7, Rue Ali Ben Ayed, Tunis, Tunisia; Military Hospital Tunis, Q5PH+896, Tunis, Tunisia; Fattouma Bourguiba University Hospital—Research Laboratory LR12SP16 and University of Monastir, QRCM+4GJ, Monastir, Tunisia; Duke-NUS Medical School, 8 College Rd, Singapore 169857, Singapore; National Heart Centre Singapore, 5 Hospital Dr, Singapore 169609, Singapore; Université Paris Cité, MASCOT Inserm Unit, 45 Rue des Saints-Pères, 75006 Paris, France; Department of Anesthesia, Burn and Critical Care Medicine, AP-HP, Hôpital Lariboisière, 2 Rue Ambroise Paré, 75010 Paris, France; Hannibal Clinic, Rue de la feuille d'Erable - les berges du lac 2, Tunis, Tunisia

**Keywords:** Echocardiography, Artificial intelligence, Task shifting

## Abstract

**Aims:**

Access to echocardiography is a significant barrier to heart failure (HF) care in many low- and middle-income countries. In this study, we hypothesized that an artificial intelligence (AI)-enhanced point-of-care ultrasound (POCUS) device could enable the detection of cardiac dysfunction by nurses in Tunisia.

**Methods and results:**

This CUMIN study was a prospective feasibility pilot assessing the diagnostic accuracy of home-based AI-POCUS for HF conducted by novice nurses compared with conventional clinic-based transthoracic echocardiography (TTE). Seven nurses underwent a one-day training program in AI-POCUS. A total of 94 patients without a previous HF diagnosis received home-based AI-POCUS, POC N-terminal pro-B-type natriuretic peptide (NT-proBNP) testing, and clinic-based TTE. The primary outcome was the sensitivity of AI-POCUS in detecting a left ventricular ejection fraction (LVEF) <50% or left atrial volume index (LAVI) >34 mL/m^2^, using clinic-based TTE as the reference. Out of seven nurses, five achieved a minimum standard to participate in the study. Out of the 94 patients (60% women, median age 67), 16 (17%) had an LVEF < 50% or LAVI > 34 mL/m^2^. AI-POCUS provided an interpretable LVEF in 75 (80%) patients and LAVI in 64 (68%). The only significant predictor of an interpretable LVEF or LAVI proportion was the nurse operator. The sensitivity for the primary outcome was 92% [95% confidence interval (CI): 62–99] for AI-POCUS compared with 87% (95% CI: 60–98) for NT-proBNP > 125 pg/mL, with AI-POCUS having a significantly higher area under the curve (*P* = 0.040).

**Conclusion:**

The study demonstrated the feasibility of novice nurse–led home-based detection of cardiac dysfunction using AI-POCUS in HF patients, which could alleviate the burden on under-resourced healthcare systems.

## Graphical Abstract

**Graphical Abstract:**
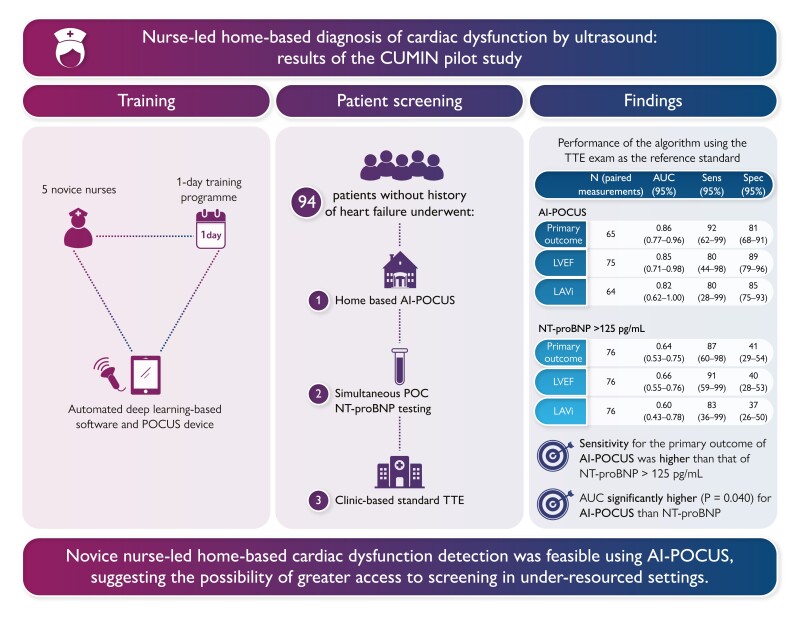

## Introduction

Globally, the prevalence of heart failure (HF) is increasing due to ageing societies and increased risk factors for HF, such as obesity, diabetes, and coronary artery disease (CAD), especially in Africa.^1^ Heart failure is a substantial public health problem in Tunisia.^2^ It has one of Africa’s oldest populations, with the highest estimated life expectancy of the continent.^2^ Timely management of HF with evidence-based pharmacotherapy can substantially improve prognosis and extend the life expectancy of a 50-year-old patient by almost 9 years.^3^ Therefore, early diagnosis is critical for initiating medical therapy.^4^

Echocardiography is the bedrock of HF diagnosis and is included in all international guidelines as a first-line investigation.^5,6^ However, the interpretation of echocardiographic images remains time-consuming and subject to high inter-and intra-reader variability and human error, even among specialists.^7,8^ Unfortunately, the number of physicians per capita in Tunisia is only half the number per capita in the USA and a quarter of the number of physicians per capita in Germany, restricting access to healthcare services such as echocardiographic investigations.^9^ Therefore, access to echocardiography is a major rate-limiting step in the HF care pathway in Tunisia. There is a critical unmet need for targeted solutions to provide more equitable access to echocardiography at a reduced cost.

Task-shifting—a situation where a qualified physician’s tasks are transferred to a health professional with a different or lower level of education^10^—can help overcome health workforce shortages in HF care delivery. Advancements in deep learning have made it possible to automate the analysis of echocardiographic images with similar accuracy as expert clinicians.^11,12^ Therefore, this pilot study aimed to estimate whether it was feasible for nurses to acquire images of sufficient quality for deep learning algorithms to interpret with acceptable accuracy. A secondary aim was to compare the accuracy of detecting an abnormal cardiac echocardiographic examination between the nurse-led artificial intelligence (AI)-point-of-care ultrasound (POCUS) examination and N-terminal pro-B-type natriuretic peptide (NT-proBNP).

## Methods

### Study design and patient population

This prospective cross-sectional study involved four centres in Tunis and one in Monastir, Tunisia. All patients were referred by a general practitioner with a suspicion of HF for an echocardiographic examination. All participants were at least 50 years old, had ≥2 cardiovascular risk factors, and had a planned cardiac echocardiographic examination performed by a senior physician certified to perform cardiac ultrasound with an indication of suspected HF. Patients participating in another clinical trial, with a previous HF diagnosis, unable or unwilling to provide informed consent, or living >30 km from the enrolling centre were excluded from the study.

Consecutive eligible patients were enrolled during a visit to the cardiologist’s office before an echocardiographic examination and asked to sign informed consent. During the screening visit, patients’ medical history and medication were recorded.

Subsequently, all participants underwent a nurse-led home-based acquisition of cardiac images by ultrasound examination within 5 days before the scheduled clinic and a cart-based echocardiographic examination. The home-based ultrasound examination was combined with a point-of-care (POC) NT-proBNP measurement. Nurses recorded patients’ height, weight, heart rate, and blood pressure during the home visit. A patient satisfaction questionnaire was administered, which included questions on whether the patient trusted the ultrasound examination performed by the nurse and the likelihood that the patient would recommend the ultrasound examination to friends or family on a scale from 1 to 10.

During the clinic echocardiographic examination, the patient underwent a standard cart-based transthoracic echocardiographic examination (TTE) by a senior cardiologist, certified to perform echocardiography, according to a standardized protocol. Patients were asked to fill in the same satisfaction questionnaire, which included questions on whether the patient trusted the ultrasound examination performed by the nurse and the likelihood that the patient would recommend the ultrasound examination to friends or family on a scale from 1 to 10.

### Ethics

This study adhered to the principles laid down in the declaration of Helsinki. Ethics approval was obtained from the Comité Local de Protection des Personnes de l’Hôpital Militaire de Tunis, Habib Thameur Hospital Teaching Ethics Committee and the Comité de Protection des Personnes du Centre.

### Point-of-care device and deep learning algorithms

We used the Torso-One cardiac probe and the Kosmos Bridge tablet with AI TRIO POCUS device.^13^ The AI TRIO provides real-time image guidance on fanning, rotating, and repositioning the probe to achieve better image quality and grades the image in real time.^13^ The AI TRIO provides real-time image guidance and grading to the nurse operator the help position the probe. The quality assessment done by the EchoNous device is based on training data. However, the hardware manufacturer has not published these results. Therefore, this information is not publicly available.

The POCUS device was combined with an automated deep learning workflow (Us2.ai, Singapore). The development and external validation of these algorithms have been published previously.^11,12^ The deep learning algorithms automatically detect the correct cardiac ultrasound views and annotate cardiac chambers to quantify parameters related to cardiac structure and function.^11,12^ A previous external validation of these algorithms showed that the automated deep-learning-based measurements of this workflow were interchangeable with human experts’ measurements.^12^

### Echocardiographic protocol and nurse training and selection

Seven nurses received 1 day of training at the beginning of the study and another one month into the study to ensure that they kept the minimal participation standard to operate the POCUS device at the Honoris Medical Simulation Center in Tunis, Tunisia. Nurses practised on healthy participants who were paid for their participation. All nurses were required to perform a minimum of 5 full examinations on patients up to a maximum of 15. Nurses who obtained three consecutive sufficient quality examinations were allowed to participate in the study. All nurses were required to record parasternal long-axis, apical four-chamber (A4C), and apical two-chamber (A2C) views. Supplementary material online, *Table S1* provides the list of parameters and views recorded by the nurse. Nurses acquired the videos and images using the POCUS device. Videos and images were subsequently uploaded to the AI-analysis software when Wi-Fi was available for further annotation and analysis. The TTE-MD reference examination was performed and interpreted by a trained cardiologist or senior physician according to conventional clinical practice in Tunisia.

### Study outcomes

The primary outcome was the sensitivity for the detection of abnormal cardiac scans, defined as a left ventricular ejection fraction (LVEF) <50% or left atrial volume index (LAVI) >34 mL/m^2^ of home-based AI-supported POC echocardiography by nurses, compared with gold standard clinic-based scans by the treating senior physicians.

Secondary outcomes included the yield, defined as the proportion of measurements obtained by the nurse-led AI-POCUS examination using the number of measurements obtained by the TTE-MD examination as the denominator, the area under the curve (AUC), specificity, positive predictive value (PPV) and negative predictive value (NPV) of the nurse-led AI-POCUS examination and a comparison of the sensitivity, specificity, AUC, PPV, and NPV of the nurse-led AI-POCUS examination with a home-based NT-proBNP text. As an exploratory outcome, we compared the AUC of the nurse-led AI-POCUS examination with an NT-proBNP measurement. A separate questionnaire assessed patient preferences during the home and clinic visits, which was designed by the investigators.

### Statistical analysis

Baseline characteristics were presented as means with standard deviation, median with interquartile range (IQR), or numbers with percentages depending on the nature and distribution of the variable. Differences among groups were examined using Student’s *t*-test, the *χ*^2^ test, or the Mann–Whitney *U* test, depending on the nature and distribution of the variables. The yield was defined as the proportion of measurements obtained by the nurse-led AI-POCUS examination using the number of measurements obtained by the conventional TTE examination as the denominator. We assessed the agreement between automated and manual measurements using Pearson’s correlation coefficient (*R*), the interclass correlation coefficient (ICC), root mean square error, and mean absolute error (MAE). The ICC was calculated using a one-way random-effects model. The AUC assessed the accuracy of the AI-enhanced nurse-led examination. The AUCs were compared between the AI-POCUS examination and the POC NT-proBNP measurement. We tabulated the sensitivity, specificity, PPV, and NPV. In addition to sensitivity and specificity, we estimated the diagnostic odds ratio to provide measures of diagnostic accuracy that are not susceptible to disease prevalence. We used Cohen’s kappa to assess the inter-rater agreement for LVEF and LAVi measurements. All analyses were performed using Stata (version 17.0, Stata Corporation, College Station, TX, USA). All statistical analyses were conducted at a significance level of 0.05, and all tests were two-tailed.

## Results

### Baseline characteristics

Seven nurses participated in the training, and five nurses passed the examination. A total of 100 patients were enrolled during the screening visit. Six patients did not attend the home-based visit or clinic visit and were lost to follow-up.

*Table 1* shows the baseline characteristics of the 94 patients who completed the home-based and clinic visits. The median age was 66.5 (IQR 59.2–72.2) years, and 56 (60%) patients were women. The median body mass index (BMI) was 27.9 (IQR 24.8–31.2) kg/m^2^. The most common signs and symptoms were reduced exercise tolerance followed by peripheral oedema and paroxysmal dyspnoea. The most common comorbidity was hypertension, followed by diabetes and CAD. The median NT-proBNP concentration during the home visit was 190.3 (IQR 105.9–7278) pg/mL, and 49 (64%) out of 76 patients with NT-proBNP measurements had an NTproBNP >125 pg/mL. Almost half of the patients were already on beta-blockers or angiotensin-converting enzyme inhibitors.

**Table 1:** Baseline characteristics

	All
*N*	94
Age, median (IQR) years	66.5 (59.2, 72.2)
Sex, *n* (%)	
Men	38 (40%)
Women	56 (60%)
**Signs and symptoms**	
Pulmonary rales, *n* (%)	7 (7%)
Shortness of breath at rest, *n* (%)	12 (13%)
Nocturnal cough, *n* (%)	8 (9%)
Reduction exercise tolerance, *n* (%)	81 (86%)
Angina, *n* (%)	8 (9%)
Peripheral oedema, *n* (%)	24 (26%)
Orthopnoea, *n* (%)	12 (13%)
Paroxysmal dyspnoea, *n* (%)	16 (17%)
**Medical history**	
Coronary artery disease, *n* (%)	11 (12%)
AF, *n* (%)	9 (10%)
Stroke, *n* (%)	5 (5%)
PAVD, *n* (%)	3 (3%)
Diabetes, *n* (%)	57 (61%)
Hypertension, *n* (%)	89 (95%)
Cancer, *n* (%)	3 (3%)
COPD, *n* (%)	7 (7%)
CKD, *n* (%)	3 (3%)
Thyroid dysfunction, *n* (%)	9 (10%)
**Laboratory**	
NT-proBNP, median (IQR)	190 (106, 728)
NT-proBNP >125 pg/mL	49 (64%)

Abbreviations: AF, atrial fibrillation; CKD, chronic kidney disease; COPD, chronic obstructive pulmonary disease; IQR, interquartile range; *N*, number of patients; NT-proBNP, N-terminal pro-brain natriuretic peptide; PAVD, peripheral arterial vascular disease.

The median TTE acquired and cardiologist-interpreted LVEF and LAVi were 61% (IQR 55–66) and 16.3 mL/m^2^ (IQR 13.1–20.9), respectively. In total, 16 patients had an LVEF <50% or LAVi >34 mL/m^2^. Among these 16 patients, 9 had a low LVEF only, 4 an enlarged LAVi only, and 3 had a low LVEF and an enlarged LAVi. Patients with a reduced LVEF or increased LAVi were more often men, had a higher prevalence of angina and atrial fibrillation, and higher median NT-proBNP concentrations than those with an LVEF ≥50% or LAVi ≤34 mL/m^2^. Out of the 81 (86%) patients with reduced exercise tolerance, 69 (85%) did not have a low EF or enlarged LAVi on the MD-TTE.

### Yield and agreement

*Table 2* shows the yield for individual parameters, which varied from a low of 63% for left ventricular internal diameter in systole to 80% for LVEF, left ventricular end-diastolic volume (LVEDV), and left ventricular end-systolic volume (LVESV). Supplementary material online, *Table S2* shows that the number of studies per nurse ranged from 1 to 29. Supplementary material online, *Table S3* shows the results of multivariable logistic regression using an interpretable LVEF or LAVi as the outcome and nurse, BMI, chronic obstructive pulmonary disease and atrial fibrillation as the independent predictors. The proportion of interpretable LVEF or LAVi was associated with differences in the nurse operator but not in participant characteristics.

**Table 2:** Agreement between automated and manual echocardiographic measurements

	Yield	*R*	ICC	RMSE	MAE	LoA
LVEDV	75 (80%)	0.75	0.67	30.7	22	14.9 ± 27.0
LVESV	75 (80%)	0.84	0.79	17.4	11.2	5.7 ± 16.5
LVEF	75 (80%)	0.71	0.68	8.6	6.6	0.9 ± 8.6
IVSd	68 (72%)	0.53	0.46	2.4	1.9	1.2 ± 2.1
LVIDd	67 (71%)	0.62	0.42	9.1	7.4	6.8 ± 6.1
LAVi	64 (68%)	0.64	0.46	10.4	8.7	7.2 ± 7.5
LVIDs	59 (63%)	0.64	0.59	7.2	5.7	1.99 ± 6.9

Abbreviations: LVEDV, left ventricular end-diastolic volume; LVESV, left ventricular end-systolic volume; LVEF, left ventricular ejection fraction; IVSd, interventricular septum thickness at end-diastole; LVIDd, left ventricular internal diameter at end-diastole; LAVi, left atrial volume index; LVIDs, left ventricular internal diameter at end-systole; LoA, limits of agreement; yield, percentage of interpretable images; *R*, correlation coefficient; ICC, intraclass correlation coefficient; RMSE, root mean square error; MAE, mean absolute error.

*Table 2* shows that the parameters showed good agreement with an ICC ranging from 0.42 for left ventricular internal diameter in diastole to 0.79 for LVESV. Supplementary material online, *Figure S1* shows the Bland–Altman graphs for LVEDV, LVEF, interventricular septal thickness, and LAVi.

### Performance of deep learning interpretation

**Figure 1:**
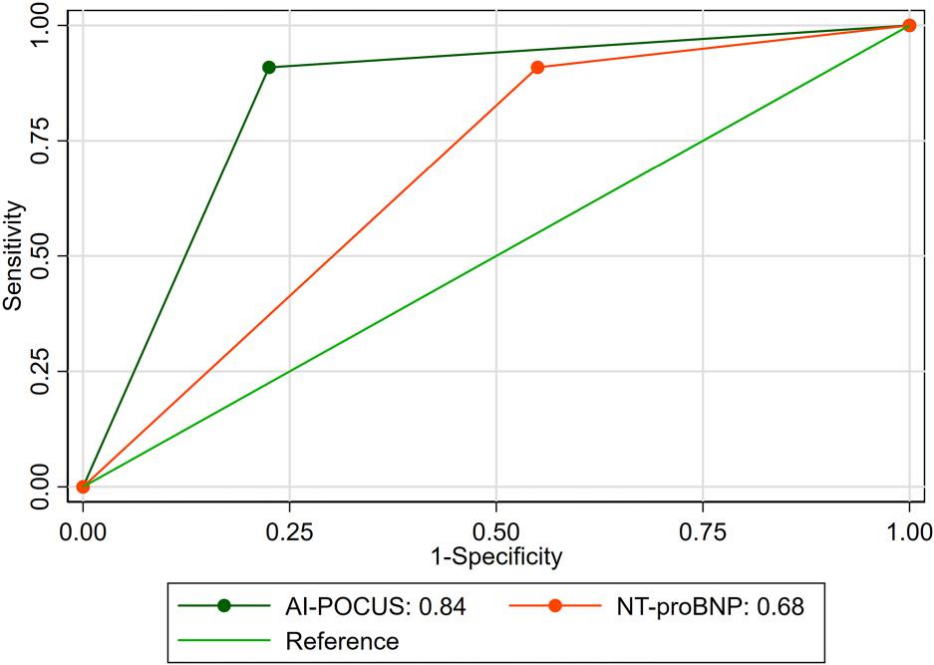
Comparative analysis of area under the curve curves. Artificial intelligence-point-of-care ultrasound refers to the home-based, nurse-led examination supported by artificial intelligence and a point-of-care ultrasound device. It is important to note that the area under the curve values depicted herein were calculated using identical samples for comparison, which may account for slight discrepancies from the area under the curves reported in *Table 3*.

*Figure 1* shows a comparison between AUC curves and *Table 3* shows the performance of the deep learning algorithms. The sensitivity for the primary outcome was 92% [95% confidence interval (CI): 62–99%]. The sensitivity for the individual components of the primary outcome was 80% (44–98%) for LVEF and 80% (28–99%) for LAVi. The diagnostic odds ratio was 51.6 (95% CI: 5.99–444.3), suggesting a good discrimination ability of AI-POCUS to identify positive cases correctly. The kappa statistic (*κ*) was 0.56 (95% CI: 0.34, 0.79).

**Table 3:** Performance of the algorithm using the transthoracic echocardiographic examination as the reference standard

	*N* ^a^	AUC (95%)	Sens (95%)	Spec (95%)	PPV (95% CI)	NPV (95% CI)
**AI-POCUS**						
Primary outcome	65	0.86 (0.77–0.96)	92 (62–99)	81 (68–91)	52 (30–74)	98 (88–99)
LVEF	75	0.85 (0.71–0.98)	80 (44–98)	89 (79–96)	53 (27–79)	97 (89–99)
LAVi	64	0.82 (0.62–1.00)	80 (28–99)	85 (75–93)	31 (10–61)	98 (89–100)
**NTproBNP >125 pg/mL**						
Primary outcome	76	0.64 (0.53–0.75)	87 (60–98)	41 (29–54)	27 (15–41)	93 (76–99)
LVEF	76	0.66 (0.55–0.76)	91 (59–99)	40 (28–53)	20 (10–34)	96 (81–99)
LAVi	76	0.60 (0.43–0.78)	83 (36–99)	37 (26–50)	10 (3–22)	96 (81–99)

Abbreviations: AI-POCUS, artificial intelligence supported point-of-care ultrasound examination; LAVi, left atrial volume index to body surface area; LVEF, left ventricular ejection fraction; NT-proBNP, N-terminal-pro hormone B-type natriuretic peptide; NPV, negative predictive value; PPV, positive predictive value.

^a^Paired measurements.

The AUC for the primary outcome was 0.86 (0.77–0.96) with a specificity of 81% (68–91%). The specificity for the individual components was 89% (79–96%) for LVEF and 85% (75–93%) for LAVi. The AUC of the nurse with AI-POCUS was higher than the AUC of NT-proBNP at a cut-off of >125 pg/mL (*P* = 0.04). The sensitivity for the primary outcome and the individual components was comparable between NT-proBNP and AI-POCUS. The specificity was higher for AI-POCUS than for NT-proBNP. Analyses stratified to A4C and A2C showed slightly better results for A2C than for A4C (see Supplementary material online, *Table S4*).

### Patient satisfaction

Supplementary material online, *Table S5* shows that patients trusted the nurse-led AI-POCUS and physician-led TTE equally. Similarly, the patients felt that the nurse and physician were comfortable performing the ultrasound examination. After the cardiologist examination, more than half of the patients preferred the nurse-led home-based AI-POCUS examination over the cardiologist-led clinic-based TTE. Only 20% of patients chose the clinic-based TTE over the home-based AI-POCUS examination.

## Discussion

This study demonstrated that novice nurses with limited training could perform home-based echocardiographic examinations and identify patients with a reduced LVEF or increased LAVi with similar accuracy as trained senior physicians in specialized clinics. Nurses acquired interpretable LVEFs and LAVis in 80% and 68% of patients, respectively, in patients’ own homes. Deep learning algorithms showed good agreement with AI-POCUS measurements with a higher accuracy for identifying patients with a reduced LVEF or increased LAVi compared with home-based POC NT-proBNP. Home-based echocardiography by nurses was well-received by patients, most of whom preferred this to traditional clinic-based echocardiography by specialists. Collectively, our results highlight the great potential of AI-POCUS to enable task-shifting of HF diagnosis from cardiologists to nurses with limited training and from clinics to the home.

Previous studies investigating the use of handheld echocardiography by novices showed different proportions of interpretable images, ranging from 78 to 97%, depending on the setting and participants.^14–16^ A fellow with 2 months of training in echocardiography could acquire 78–85% technically adequate images with a handheld device.^16^ A study investigating the diagnostic accuracy of handheld scans by inexperienced medical residents found that only 2 (3%) out of 72 examinations yielded a non-interpretable LVEF.^14^ Similarly, a study in Spain found that only 7 out of 223 examinations were performed using a handheld scanner by an internal medicine resident who received limited training.^15^ However, in a study by Aldaas *et al*.,^14^ all examinations were performed in a controlled hospital setting just after a practice session under the supervision of skilled sonographers. In a study by López-Palmero *et al*.,^15^ internal medicine residents received a 70 h course, which was substantially longer than our one-day course. Importantly, in our study, novices performed the echocardiographic examination in the patients’ homes without external supervision. We found that the nurse operator was the only predictor of an interpretable LVEF or LAVi in our study, suggesting that careful selection and subsequent training might improve the proportion of interpretable LVEFs and LAVis.

The nurse-led AI-POCUS examination had a high sensitivity for identifying patients with a reduced LVEF or increased LAVi and showed good agreement with expert-measured TTE values. A recent meta-analysis of the diagnostic value of handheld ultrasound devices to diagnose a reduced LVEF found a pooled sensitivity of 83% (95% CI: 71–90%).^17^ It is perhaps unsurprising that AI-POCUS outperformed NT-proBNP for diagnosing structural changes. Our results showed higher sensitivities, suggesting that AI software can further augment the use of POCUS devices by novices. Previous validation studies showed good automated LVEF and LAVi measurement agreement in various external validation cohorts with TTE studies.^11,12^ Only 16% of patients had an increased LAVi or decreased LVEF, which could be explained by the relatively young age of the cohorts. The higher sensitivity of AI-POCUS compared with that of NT-proBNP suggests that AI-POCUS might be considered a first-line examination to rule out patients without HF in a primary care or home-based setting. However, practical implementation would require adding additional measurements relating to HF aetiology, including valve disease and regional wall motion abnormalities. Importantly, our results suggest that patients preferred a home-based AI-POCUS investigation over a clinic-based TTE examination.

Our study results highlight the possibility of integrating nurse-led home-based AI-supported ultrasound examinations in remote home-based HF monitoring. Previous studies demonstrated that remote management of patients with HF can reduce mortality, morbidity, and costs.^18–22^ For example, the recent MONITOR-HF trial showed that haemodynamic monitoring with the CardioMEMS device improved the quality of life and reduced HF hospitalizations in patients with moderate-to-severe HF. Similar strategies can be expanded by incorporating nurse-led home-based echocardiographic examinations to monitor patients and adjust treatment at an earlier stage when the condition of patients worsens. However, other parameters will need to be included, including inferior vena cava imaging.

Our results suggest that home-based nurse-led AI-POCUS investigations might supplement the HF care pathway in Tunisia. AI-POCUS is best positioned at the beginning of the HF care pathway to triage patients who might require further investigation. A previous study investigating the use of POCUS to prevent inappropriate TTE requests suggested that handheld ultrasound could be an effective gatekeeper for TTE studies.^23^ Combined with improved training and enhanced standardization of novices’ AI-POCUS examinations can help reduce the workload on busy echocardiography laboratories in Tunisia and can increase access to cardiac ultrasound examinations.

### Limitations

This study had various limitations. First, this was a pilot study with a limited sample size. The specific setting in Tunisia might preclude generalization of our results to countries or healthcare settings in other countries or regions. Images were not separately analysed for image quality. Since CUMIN’s primary aim was to estimate the sensitivity of nurse-led AI-POCUS for diagnosing a LVEF reduction or increased LAVi, we did not capture other diagnoses that might be missed in the case report form. This study included only a few patients with atrial fibrillation. Therefore, we could not stratify our results to those with and without atrial fibrillation. Nurses could not acquire sufficient quality images in all patients, which did not allow us to compare patients without AI-POCUS measurements with patients with MD-TTE measurements.

## Conclusions

Our study demonstrated the feasibility of novice nurse–led home-based cardiac identification of a reduced LVEF or increased LAVi using AI-POCUS. Such task-shifting approaches, from overloaded clinical echocardiography laboratories to home HF nurses with limited training, might help reduce the burden of resource utilization in the Tunisian healthcare system.

## Supplementary material

Supplementary material is available at *European Heart Journal – Digital Health*.

## Supplementary Material

ztad079_Supplementary_Data

## Data Availability

The datasets generated during and/or analysed during the current study are not publicly available because participants did not consent to their data being shared publicly but are available from the corresponding author on reasonable request for reproducing the results.
