# Supplementary material for: Nurse-led home-based detection of cardiac dysfunction by ultrasound: results of the CUMIN pilot study
Source: Eur Heart J Digit Health. 2023 Dec 12;5(2):163–9. doi: 10.1093/ehjdh/ztad079 (PMC10944680; doi:10.1093/ehjdh/ztad079)

**Supplementary material.**

*Supplementary Table 1:* Overview of views acquired by the nurse and parameters measured by the deep-learning algorithms.

|  | **View** | **Measurements (primary)** | **Calculated (IF primaries are present)** |
| --- | --- | --- | --- |
| Primary | **PLAX** | IVSd |  |
|  |  | LVIDd |  |
|  |  | LVIDs |  |
| Primary | **A4C** | LVEDV MOD (Singleplane) |  |
|  |  | LVESV MOD (Singleplane) |  |
|  |  | LVEF MOD (Singleplane) |  |
|  |  | LAESV MOD (Singleplane) |  |
| Optional | **A2C** | LVEDV MOD (Singleplane) | *LVEDV MOD (biplane)* |
|  |  | LVESV MOD (Singleplane) | *LVEDV MOD (biplane)* |
|  |  | LVEF MOD (Singleplane) | *LVEF MOD (biplane)* |
|  |  | LAESV MOD (Singleplane) | *LAESV (MOD (biplane)* |

*Abbreviations:* A2C, Apical Two-Chamber View; A4C, Apical Four-Chamber View; IVSd, Interventricular Septum thickness at end-diastole; LAESV, Left Atrial End-Systolic Volume; LVEDV, Left Ventricular End-Diastolic Volume; LVEF, Left Ventricular Ejection Fraction; LVESV, Left Ventricular End-Systolic Volume; LVIDd, Left Ventricular Internal Diameter at end-diastole; LVIDs, Left Ventricular Internal Diameter at end-systole; MOD, Modified Simpson’s Method; PLAX, Parasternal Long Axis View; RAarea, Right Atrial Area; RVIDd, Right Ventricular Internal Diameter at end-diastole.

*Supplementary Table 2:* Number of acquired studies per nurse.

| **Nurse** | **Number** |
| --- | --- |
| RN1 | 16 |
| RN2 | 1 |
| RN3 | 29 |
| RN4 | 22 |
| RN5 | 26 |
| Total | 94 |

*Abbreviations:* RN, research nurse

*Supplementary Table 3***:** Results of multivariable logistic regression

|  | **LVEF** | **LAVi** |
| --- | --- | --- |
|  | Odds ratio (95%CI) | Odds ratio (95%CI) |
| Age | 1.03 (0.96-1.11) | 1.04 (0.98-1.11) |
| Women | 0.71 (0.19-2.65) | 0.60 (0.21-1.72) |
| Nurse |  |  |
| 1 | ref | ref |
| 2 | NA | NA |
| 3 | 1.77 (0.43-7.30) | 1.62 (0.42-6.31) |
| 4 | 15.84 (1.64-152.36) | 6.02 (1.21-30.08) |
| 5 | 23.14 (2.32-231.11) | 5.16 (1.19-22.24) |
| COPD | 1.17 (0.08-17.01) | 2.33 (0.19-29.00) |
| BMI | 0.99 (0.87-1.12) | 1.02 (0.92-1.14) |
| AF | 0.46 (0.06-3.46) | 1.13 (0.19-6.66) |

*Abbreviations:* AF, Atrial Fibrillation; BMI, Body Mass Index; COPD, Chronic Obstructive Pulmonary Disease; LAVi, Left Atrial Volume Index; LVEF, Left Ventricular Ejection Fraction; NA, Not Applicable.

*Supplementary Table 4*: Analyses stratified to A2C and A4C

|  | **AUC (95%)** | **Sens (95%)** | **Spec (95%)** |
| --- | --- | --- | --- |
| **A4C** |  |  |  |
| LVEF | 0.70 (0.55-0.84) | 54 (25-81) | 86 (74-93) |
| LAVi | 0.62 (0.52-0.72) | 31 (14-52) | 94 (78-99) |
| **A2C** |  |  |  |
| LVEF | 0.79 (0.63-0.95) | 70 (45-93) | 88 (76-96) |
| LAVi | 0.70 (0.57-0.83) | 47 (23-72) | 93 (77-99) |

*Supplementary Table* 5: Patient experiences

|  | **Nurse visit** | **Cardiologist visit** |
| --- | --- | --- |
| **Will you trust the results of the exam** |  |  |
| Not at all | 1 (1%) | 1 (1%) |
| Rather no | 0 | 1 (1%) |
| Rather yes | 8 (9%) | 6 (8%) |
| Absolutely yes | 85 (90%) | 71 (90%) |
| **Is the operator comfortable with ultrasound?** |  |  |
| Not at all | 0 | 1 (1%) |
| Rather no | 1 (1%) | 1 (1%) |
| Rather yes | 2 (2%) | 5 (6%) |
| Absolutely yes | 91 (97%) | 72 (91%) |
| **If you need to redo this exam and are given a choice, will you do it by the nurse at home or the cardiologist at the hospital?** |  |  |
| NA |  | 15 (16%) |
| Hospital |  | 19 (20%) |
| Either |  | 12 (13%) |
| Home |  | 48 (51%) |
| **On a scale of 1 (Low) to 10 (High), how likely would you recommend a home-based examination to a friend or family member?** | 10 (9, 10) | 9 (8, 10) |

*Abbreviations:* NA, not applicable

*Supplemental Figure 1:* Bland-Altman plots for LVEDV, LVEF, Interventricular Septal Thickness and LAVi. Abbreviations: LVEDV, Left Ventricular End-Diastolic Volume; LVEF, Left Ventricular Ejection Fraction; LAVi, Left Atrial Volume Indexed to Body Surface Area.


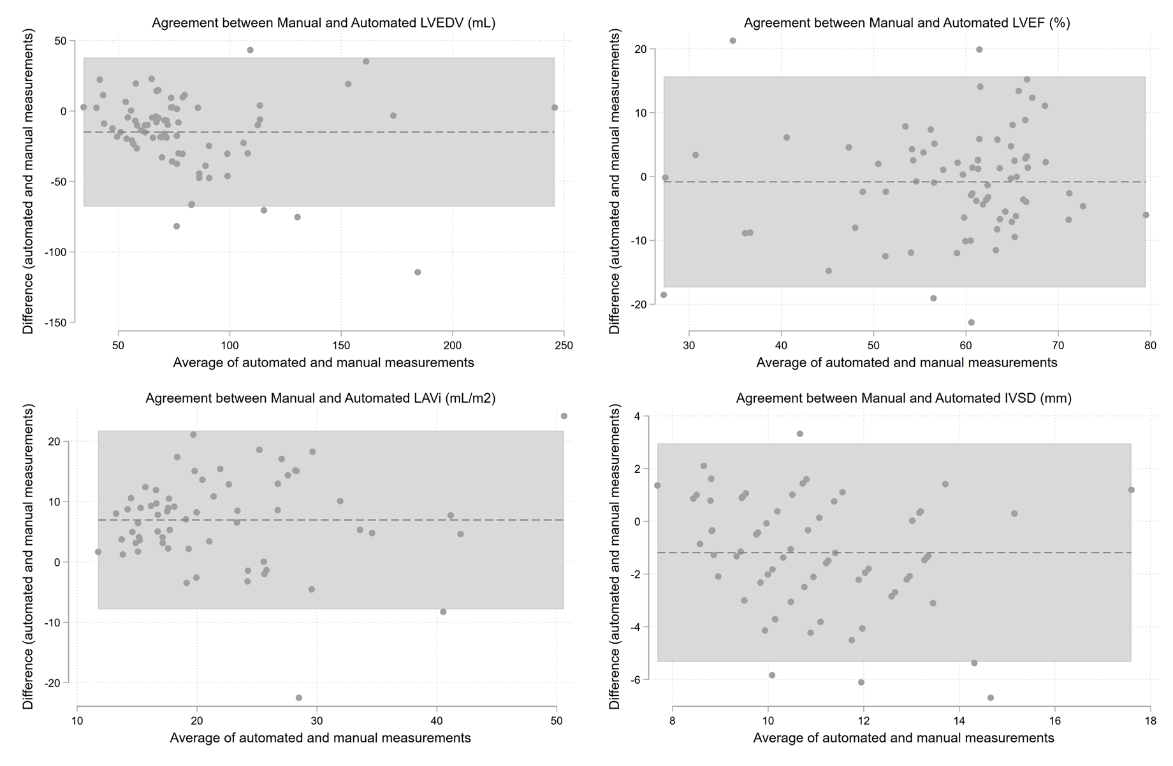

Supplement: ztad079_Supplementary_Data [file ztad079_supplementary_data.zip › 2023.11.6 Supplementary material.docx]
